# Editorial: Secondary traumatic stress: Risk factors, consequences, and coping strategies

**DOI:** 10.3389/fpsyg.2023.1148186

**Published:** 2023-02-28

**Authors:** Daniela Acquadro Maran, Valentina Dolce, Lara Colombo

**Affiliations:** ^1^Department of Psychology, University of Turin, Turin, Italy; ^2^Institut de Psychologie, Université Lumière Lyon 2, Lyon, France

**Keywords:** secondary trauma stress, coping strategies, risk factors, consequence, vicarious trauma

The concept of “vicarious traumatization” was first introduced by McCann and Pearlman (1990), and from then on the construct gained increasing interest among researchers (Branson, 2019; Ashley-Binge and Cousins, 2020). Figley (1995) used the term “secondary traumatic stress” or “vicarious traumatization” to refer to all those individuals who, because of repeated and close relationships with individuals who have directly experienced traumatic events, are at risk of becoming indirect victims of the same trauma and experiencing forms of emotional decompensation. Everyone is potentially exposed to at least one traumatic event during their lifetime; for people in helping professions, the likelihood increases because they themselves and others are exposed to more stressful events (Argentero and Setti, 2011). Thus, it can be said that this risk affects all professions that provide assistance and support to individuals and/or populations who are victims of trauma (Figley, 1999).

Concurrent with the expansion of the topic into professional research, a meta-analysis was conducted to develop Australian clinical guidelines for working with adult survivors of child abuse (Adult Survivors of Child Abuse, 2012). Adult Survivors of Child Abuse (2012) recognizes that working with traumatized individuals through both direct and indirect means requires significant commitment and notes that knowledge of the risk factors associated with these forms of abuse is insufficient to effectively deal with situations that result in severe distress. Furthermore, it is increasingly recognized that secondary traumatic stress is a common, natural, and potentially harmful response for all professionals working with individuals affected by traumatic events (Sprang et al., 2019). Setti and Argentero (2012) identify vicarious traumatization as a form of occupational stress and, consequently, a psychosocial risk factor characteristic of all helping professions.

In terms of consequences, psychological symptoms are similar to those of post-traumatic stress disorder acquired through contact with people suffering from the effects of trauma. Thus, workers may experience negative consequences such as anxiety, depression, sleep disturbances, intrusive thoughts, maladaptive coping strategies (e.g., increased use of psychotropic substances), and negative emotions such as anger and feelings of inadequacy (Collins and Long, 2003). In addition, secondary trauma can impact resilience and affect worker performance: e.g., decreased effectiveness of intervention with patients/clients/citizens, etc., inability to express negative feelings through denial of difficulties at work, emotional withdrawal that can impact emotional (relatives, friends) and social (colleagues, supervisors) environments.

This Research Topic includes four research articles that deepen our understanding of the phenomenon, its analysis, and ways to alleviate it.

Three articles are from China and one from Slovakia. Wu et al. conducted a study on the coping style choices of recruits under psychological stress while performing military tasks. One thousand and twenty-eight Chinese recruits were interviewed in two waves of survey. Results showed that recruits' psychological stress negatively affected positive coping styles and positively correlated with negative coping styles. In addition, self-efficacy and social support mediated the relationship between psychological stress and positive coping styles, and self-efficacy mediated the relationship between psychological stress and negative coping styles. More importantly, self-efficacy and social support formed the mediating chain between psychological distress and positive coping styles. The use of appropriate coping strategies can help reduce stress and improve task performance through their effect on self-efficacy ratings.

Coping style was also analyzed in the study by Sun et al. The authors examined the factors and mechanisms that influence depression in medical professions. In their cross-sectional study, 1,139 physicians were surveyed using a cluster sampling method. Questionnaires included the Psychological Capital Questionnaire, the Chinese Employees' Organizational Commitment Questionnaire, the Coping Style Questionnaire, and the Depression Self-Rating Scale. The study found that 41.6% of the physicians suffered from depression. Among them, 17.0% of the physicians suffered from moderate depression and 2.6% from severe depression. Results showed that psychological capital was sequentially associated with higher organizational commitment and a more positive coping style, which led to a reduction in depression among physicians.

Yan et al. examined the psychometric properties of a simplified version of the Secondary Trauma Questionnaire for Chinese on a potentially traumatized sample (*N* = 875) of physicians, nurses, teachers, administrators, and social workers. Results showed that the full scale had good internal consistency, convergent validity, discriminant validity, and factorial validity. The CFA confirmed a single-factor structure; the configural, metric, scalar, and strict invariance of the secondary trauma questionnaire were acceptable for all genders. The present results suggest that the scale is a reliable and valid self-report instrument for use with potentially traumatized individuals in China and support the notion that it is suitable for further cross-cultural adaptation. Examining the phenomenon in multiple cultures or measuring the differences between cultures could be useful to better understand the process underlying the experience and its consequences.

In their study, Halamová et al. from Slovakia examined the short- and long-term effectiveness of a novel emotion-focused training for helping professions on levels of compassion fatigue (secondary traumatic stress and burnout), self-criticism, self-compassion, and compassion for others. A randomized controlled trial was conducted with 253 participants. Results showed that participants in the experimental group reported significantly lower scores for secondary traumatic stress, burnout, and self-criticism and higher scores for self-compassion at the conclusion of the intervention, and that these results persisted 2 months after the conclusion of the intervention. Compared with the control group, participants in the experimental group had significantly lower scores for secondary traumatic stress, burnout, and self-criticism and higher scores for self-compassion after the intervention. The training proved effective in reducing compassion fatigue (secondary traumatic stress and burnout) and self-criticism and increasing self-compassion.

## Author contributions

DA, VD, and LC contributed substantially and intellectually to the Research Topic and approved the editorial for publication. All authors contributed to the article and approved the submitted version.

## References

[B1] Adult Survivors of Child Abuse. (2012). Practice Guidelines for Treatment of Complex Trauma and Trauma Informed Care and Service Delivery, eds C. A. Kezelman and P. A. Stavropoulous Sydney, NSW: ASCA.

[B2] Argentero P. Setti I. (2011). Engagement and vicarious traumatization in rescue workers. Int. Arch. Occup. Environ. Health 84, 67–75. 10.1007/s00420-010-0601-8 21079988

[B3] Ashley-Binge S. Cousins C. (2020). Individual and organisational practices addressing social workers' experiences of vicarious trauma. Practice 32, 191–207. 10.1080/09503153.2019.162020131828329

[B4] Branson D. C. (2019). Vicarious trauma, themes in research, and terminology: a review of literature. Traumatology 25, 2–10. 10.1037/trm0000161

[B5] Collins S. Long A. (2003). Working with the psychological effects of trauma: consequences for mental health-care workers – a literature review. J. Psychiatr. Mental Health Nurs. 10, 417–424. 10.1046/j.1365-2850.2003.00620.x 12887633

[B6] Figley C. R. (1995). “Systemic traumatization: secondary traumatic stress disorder in family therapists,” in Integrating Family Therapy: Handbook of Family Psychology and Systems Theory, eds R. H. Mikesell, D.-D. Lusterman, and S. H. McDaniel (Washington, DC: American Psychological Association), 571–581. 10.1037/10172-033

[B7] Figley C. R. (1999). “Police compassion fatigue (PCF): theory, research, assessment, treatment, and prevention,” in Police Trauma: Psychological Aftermath of Civilian Combat, eds J. M. Violanti and D. Paton (Baltimore: Charles C Thomas Publisher, Ltd.), 37–53.

[B8] McCann I. L. Pearlman L. A. (1990). Vicarious traumatization: a framework for understanding the psychological effects of working with victims. J. Traumat. Stress 3, 131–149. 10.1007/BF00975140

[B9] Setti I. Argentero P. (2012). Vicarious Trauma: A Contribution to the Italian Adaptation of the Secondary Traumatic Stress Scale in a Sample of Ambulance Operators. Firenze: Giunti Organizzazioni Speciali. 10.1037/t21882-000

[B10] Sprang G. Ford J. Kerig P. Bride B. (2019). Defining secondary traumatic stress and developing targeted assessments and interventions: lessons learned from research and leading experts. Traumatology 25, 72. 10.1037/trm0000180

